# Child ADHD and autistic traits, eating behaviours and weight: A population‐based study

**DOI:** 10.1111/ijpo.12951

**Published:** 2022-06-24

**Authors:** Holly A. Harris, April Bowling, Susana Santos, Kirstin Greaves‐Lord, Pauline W. Jansen

**Affiliations:** ^1^ Department of Child & Adolescent Psychiatry/Psychology Erasmus MC, University Medical Center Rotterdam The Netherlands; ^2^ Generation R Study Erasmus MC, University Medical Center Rotterdam The Netherlands; ^3^ Department of Public Health and Nutrition Merrimack College, School of Health Sciences North Andover Massachusetts USA; ^4^ Department of Psychiatry University of Massachusetts Chan Medical School Worcester Massachusetts USA; ^5^ Department of Pediatrics Erasmus MC – Sophia Children's Hospital, University Medical Center Rotterdam Rotterdam The Netherlands; ^6^ Department of Psychology University of Groningen Groningen The Netherlands; ^7^ Autisme Team North‐Netherland Jonx part of Lentis Psychiatric Institute Groningen The Netherlands; ^8^ Department of Psychology, Education & Child Studies Erasmus University Rotterdam Rotterdam The Netherlands

**Keywords:** appetitive traits, attention‐deficit/hyperactivity disorder traits, autistic traits, BMI, cohort, eating behaviour

## Abstract

**Background:**

Children with Autism Spectrum Disorder (ASD) and Attention‐Deficit/Hyperactivity Disorder (ADHD) have an increased obesity risk. Although these conditions commonly co‐occur, shared factors relating to obesity risk are unknown.

**Objectives:**

To examine the shared and unique associations of ADHD and autistic traits with eating behaviours and BMI.

**Methods:**

Children (*N* = 4134) from the population‐based Generation R Study were categorized into subgroups based on parent‐reported ADHD and autistic traits scores at 6 years: ADHD_High_, ASD_High_, ADHD+ASD_High_ and REF (reference group: ADHD+ASD_Low_). Multiple linear regressions examined the associations between subgroups and eating behaviours (at 10 years) and BMIz (at 14 years), relative to REF. Mediation analyses tested the indirect effect of subgroup and BMIz through eating behaviours.

**Results:**

ADHD + ASD_High_ children expressed both food approach (increased food responsiveness and emotional overeating) and avoidant eating behaviours (increased emotional undereating, satiety responsiveness/ slowness in eating and picky eating, and decreased enjoyment in food). ASD_High_ children were more food avoidant, while ADHD_High_ children had more food approach behaviours and greater BMIz. ADHD_High_ and BMIz were indirectly associated with food responsiveness and emotional overeating.

**Conclusions:**

ADHD and autistic trait phenotypes show distinct associations with potential obesity risk factors, and further research is needed to improve targeted early intervention.

## 1 INTRODUCTION

Children with neurodevelopmental disorders face unique challenges for maintaining healthy lifestyle behaviours^1^ and have a disproportional risk of developing eating problems^2^ and obesity^3^, ^4^ compared to neurotypical children. Two common neurodevelopmental disorders are Autism Spectrum Disorders (ASD) and Attention‐Deficit/Hyperactivity Disorder (ADHD). Features of ASD include social communication deficits, and restrictive and repetitive behavioural patterns; while features of ADHD include impaired levels of attention, disorganization and/or hyperactivity.^5^ Although ASD and ADHD are distinct conditions, they frequently co‐occur and demonstrate evidence of shared aetiology.^6^ Up to half of the children with ASD also fulfil ADHD diagnostic criteria.^7^, ^8^ Moreover, those children with ASD who do not fulfil the ADHD diagnostic criteria may still have elevated (yet subclinical) ADHD traits, and vice versa.^6^ Research to date has primarily examined obesity risk factors in case–control studies or clinical samples of children with either an ADHD or ASD diagnosis, often excluding children with a co‐morbid diagnosis.^9^ At the population level, ADHD and autistic traits exist on a continuum, and both traits have been independently associated with poor diet quality.^10^, ^11^ However, the independent or synergistic effects of ADHD and autistic traits on obesity risk factors, like eating behaviours, are unknown.

Eating behaviours (or ‘appetitive traits’) describe appetitive predispositions and sensitivity to environmental food cues, and thus play a role in childhood obesity risk.^12^ Dimensions of eating behaviours have been conceptualized as ‘food approach’ or ‘food avoidant’,^13^ which associate positively and negatively with child weight, respectively.^12^ Children with ASD tend to express more food avoidant behaviours such as food selectivity or picky eating, where only a small variety of foods are consumed.^14^ Picky eating is linked to lower Body Mass Index (BMI) in the general population.^15^ However, autistic traits may modulate this association to predict higher BMI,^16^ perhaps through ‘selective overeating’ of a small variety of food.^17^ This suggests that other eating behaviours beyond picky eating (or other food avoidant traits) may play a role in ASD‐obesity risk. Owing to the common co‐occurrence of ASD and ADHD,^7^, ^8^ it is critical to explore the association between autistic traits and other eating behaviours in the context of ADHD traits. Evidence pertaining to associations between ADHD traits and eating behaviours are inconsistent. One population‐based study showed that ADHD traits were cross‐sectionally associated with increased food approach behaviours like food responsiveness and emotional overeating, but there was limited evidence for associations with food avoidant behaviours.^18^ In contrast, another population‐based cohort showed a small positive correlation between ADHD traits and satiety responsiveness, but no association with food responsiveness, emotional overeating or enjoyment of food.^19^ To assist caregivers (often parents) and practitioners in obesity prevention efforts, it is important to understand the risk mechanisms of how ADHD and autistic traits may co‐act or be separately associated with eating behaviours, and consequently, weight.

Therefore, this population‐based study aims to examine the shared and unique associations of elevated ADHD and autistic traits at child age 6 years, and eating behaviours at 10 years. A second aim was to examine the association of ADHD and autistic traits at 6 years with BMIz score at 14 years, and explore whether this is mediated by eating behaviours at 10 years.

## 2 METHODS

### 2.1 Study design and population

The study is embedded in the Generation R Study (Generation R), a population‐based cohort on health and development from fetal life onwards. All pregnant women living in Rotterdam, the Netherlands, with an expected delivery date between April 2002 and January 2006 were invited to participate (participation rate: 61%). The study was conducted in accordance with the guidelines proposed in the World Medical Association Declaration of Helsinki and has been approved by the Medical Ethical Committee of Erasmus Medical Center Rotterdam. Written informed consent was obtained from parents of all children. Full consent for participation in phase 3 of Generation R (covering child age 6 and 10 years, *n* = 7254) was obtained from children and their parents. Children with full information on ADHD and autistic traits at 6 years and appetitive traits at 10 years were included in the analyses for aim 1 (*n* = 4314). Compared to children included in the analyses, those excluded due to missing values (*n* = 2940), more often had a non‐Western background, a lower birthweight, and a higher BMIz at 14 years; their mothers were younger, had lower levels of education, and higher psychopathology symptom scores (all *p* ≤ 0.001). The second aim consisted of a subsample of *n* = 3495 children who had additional information available on BMIz at 14 years (see Figure S1 for participant flow chart).

### 2.2 Measures

#### 2.2.1 ADHD and autistic traits

Parents (93% mothers) completed the well‐validated Child Behaviour Checklist (CBCL)/1.5–5^20^ at child age 6 years old, from which two subscales were used to assess ‘ADHD traits’ and ‘autistic traits’. The DSM (Diagnostic and Statistical Manual of Mental Disorders)‐Oriented ‘Attention deficit/hyperactivity problems’ subscale assessed ADHD traits (6 items, e.g., ‘Cannot sit still, is restless or hyperactive’). The DSM‐Oriented ‘Pervasive developmental problems’ subscale assessed autistic traits (13 items, e.g., ‘Disturbed by any change in routine’). Parents responded to items on a 3‐point Likert Scale from ‘0’ (*Never*) to ‘2’ (*Often*) in the past month. Items were summed to produce an overall sum score for each subscale. The internal reliability for each subscale was acceptable (ADHD traits *α* = 0.78; autistic traits *α* = 0.72).

The optimal approach to examining the shared and unique characteristics of ASD and ADHD in population‐based cohorts is highly debated.^21^ Therefore, we took a parsimonious approach to categorize children based on a combination of ADHD and autistic traits.^20^ A similar approach has been undertaken before in the current cohort.^22^ First, binary variables were created to indicate ‘high’ (clinical or significant sub‐clinical; ≥80th percentile) or ‘low’ (non‐significant sub‐clinical;<80th percentile) ADHD trait score. For descriptive purposes, the proportion of children in the 80th percentile cut‐points were compared against more specific screening tools for ADHD and autism to determine clinical significance of these cut‐points in the sample, where data was available. At child age 8 years, parents completed the Conners' Parent Rating Scale–Revised Short Form (CPRS‐R:S), a 27‐item questionnaire that screens for ADHD.^23^ Of those children in our study sample with a clinically significant score (*t* > 65) on the CPRS‐R:S ‘Hyperactivity’ scale, 73% also had ‘high’ ADHD traits (≥80th percentile) on the CBCL.^24^ At child age 6 years, parents completed the 18‐item short‐form of the Social Responsiveness Scale,^25^ which is a screening questionnaire providing a valid measure of (sub)clinical autistic traits. Based on sex‐specific screening cutoffs recommended in population‐based settings, *n* = 51 of children in our sample met the clinical cutoff.^26^ Of those children, the proportion of children who had ‘high’ ASD traits (≥80th percentile) on the CBCL was 92% (*n* = 47). Therefore, using the binary scores derived from the 80th percentile CBCL cut‐points, children were then categorized into four subgroups based on these binary variables: high ADHD traits (ADHD_High_, ≥80th percentile on ADHD traits and <80th percentile on autistic traits), high autistic traits (ASD_High_, <80th percentile on ADHD traits and ≥80th percentile on autistic traits), high ADHD and autistic traits (ADHD+ASD_High_, ≥80th percentile on ADHD and autistic traits) and a reference group of children with low ADHD and autistic traits (REF, <80th percentile on both ADHD and autistic traits).

#### 2.2.2 Eating behaviours

At child age 10 years, parents completed six subscales from the widely‐used Children's Eating Behaviour Questionnaire (CEBQ).^13^ The CEBQ has demonstrated good psychometric properties,^13^ and ecological validity in behavioural tests.^27^, ^28^ Five subscales from the CEBQ were examined, three of which captured ‘food approach’ behaviours: food responsiveness (5 items, e.g., ‘My child is always asking for food’), enjoyment of food (4 items, e.g., ‘My child loves food’) and emotional overeating (4 items, e.g., ‘My child eats more when anxious’); and two that captured ‘food avoidant’ behaviours: emotional undereating (4 items, e.g., ‘My child eats less when (s)he is upset’) and satiety responsiveness/slowness in eating (9 items, e.g., ‘My child gets full before his/her meal is finished’), hereafter referred to as satiety responsiveness. Also at 10 years, parents completed the Stanford Feeding Questionnaire,^29^ to assess picky eating. This scale (4 items, e.g., ‘My child eats a limited number of types of food’) is conceptually similar to the food fussiness subscale of the CEBQ. This subscale has been validated in a population‐based Danish cohort^29^ and the items used in current study show good construct validity with parent‐reported child picky eating at 11 years old.^30^ Items for each of the subscales were measured on a 5‐point Likert scale from ‘1’ (never) to ‘5’ (always) and averaged to produce a mean subscale score. All child eating subscales used in the current study showed good internal reliability (*α* = 0.84–0.92). Z‐scores for each subscale were created to facilitate effect size comparisons.

#### 2.2.3 BMIz score

Children's height and weight were measured by research assistants at the researcher center visit at age 14 years. Height and weight were measured without shoes and heavy clothing. Height was measured to the nearest millimetre by a stadiometer (Holtain Limited, Dyfeld, UK). Weight was measured to the nearest gram using an electronic scale (SECA, Almere, The Netherlands). BMI was calculated [weight (kg)/height (m^2^)] and sex‐ and age‐adjusted BMIz score was obtained based on Dutch reference growth curves^31^ processed via the Growth Analyser program (Growth Analyser 4.0, Dutch Growth Research Foundation, Rotterdam, The Netherlands).

#### 2.2.4 Covariates

Several possible confounders are considered in the analyses based on previous studies.^32^, ^33^ Information on child sex and birth weight was obtained from hospital/midwife registries. Child ethnicity (categorized as Western or non‐Western) was based on the country of birth of both biological parents, which was assessed at enrollment. Information on child BMIz was also collected at age 6 years using the same methods described in the paragraph above. Information on ADHD medication use was collected at 10 years, with *n* = 113 children reported to be on ADHD medication. Maternal age, education level and global psychopathology were assessed by postal questionnaire during pregnancy. Psychopathology was assessed with the Brief Symptom Inventory.^34^

### 2.3 Statistical analyses

All analyses were performed in R software (R Foundation for Statistical Computing, Vienna, Austria). Two‐sided statistical significance was set as *α* < 0.05. Sample characteristics were described for the whole sample and stratified by child ADHD/autistic trait subgroup.

#### 2.3.1 Aim 1

Multiple linear regression models were run to examine the unique and combined effects of elevated ADHD and autistic traits on eating behaviour within the sample. Dummy coded variables for the ADHD and autistic traits subgroups (ADHD_High_, ASD_High_, ADHD + ASD_High_ and REF) were entered as the independent variables and separate models were run for each eating behaviour as the outcome variables. Each model controlled for covariates. Multiple imputation on missing covariates was performed in the MICE^35^ package using 20 imputed datasets to obtain pooled estimates from the multiple linear regression models. The Benjamini‐Hochberg False Discovery Rate was applied to correct for multiple testing. Effect modification by child sex was also examined. As a supplemental analysis, we examined the independent effect of ADHD and autistic traits as continuous variables on eating behaviours in multiple linear regression analyses. First, the individual effect of ADHD traits on eating behaviour was examined, unadjusted for autistic traits (model 1). Next, the individual effect of autistic trait score on eating behaviour was examined, unadjusted for ADHD traits (model 2). ADHD and autistic traits scores were then entered into a final model together to investigate whether these factors were independently associated with eating behaviour (model 3).

#### 2.3.2 Aim 2

The unique and combined effects of ADHD and autistic traits on BMIz were examined using a similar procedure to Aim 1; ADHD and autistic traits subgroups were included as the independent variable and BMIz at 14 years as the outcome variable. As a preliminary step to the mediation analysis, multiple linear regressions were run to examine the association between each eating behaviour at 10 years and BMIz at 14 years. Mediation analysis was performed using the *lavaan* package^36^ to estimate the indirect effects of ADHD and autistic traits subgroup on BMIz at 14 years through eating behaviours, adjusting for covariates. Only the subgroups (independent variable) and eating behaviours (mediators) associated with BMIz were examined in the mediation analysis. Maximum likelihood estimation was used to obtain the bootstrapped estimates (with 5000 resamples) and 95% Confidence Intervals (CI) for the indirect, total and direct effects. Missing values on covariates were imputed using Full Information Maximum Likelihood.^37^ Analyses were rerun without imputed data on covariates as a sensitivity analysis.

## 3 RESULTS

Sociodemographic characteristics of the full sample and stratified by subgroup are shown in Table 1. At 6 years, ADHD and autistic traits were positively correlated (*r* = 0.44, *p* < 0.001).

**TABLE 1:** Imputed population characteristics and descriptives on the main variables of interest^a^ for the study sample, and by ADHD and autistic traits subgroup^b^

Child	All *n* = 4314	REF *n* = 2806 (65.0%)	ADHD_High_ *n* = 632 (14.6%)	ASD_High_ *n* = 456 (10.6%)	ADHD + ASD_High_ *n* = 420 (9.7%)
Boys (%)	2141 (49.6)	1269 (45.2)	354 (56.0)	258 (56.6)	260 (61.9)
Birth weight (g), mean ± SD	3430.6 ± 572.3	3447.0 ± 563.9	3391.8 ± 574.1	3448.5 ± 569.1	3360.3 ± 622.0
Western ethnicity (%)	3388 (78.5)	2267 (80.8)	478 (75.6)	371 (81.4)	272 (64.8)
ADHD medication (%)	141 (3.3)	48 (1.7)	43 (6.8)	10 (2.2)	40 (9.5)
BMI*z* ^c^, mean ± SD					
6 years	0.19 ± 0.87	0.18 ± 0.85	0.24 ± 0.93	0.14 ± 0.89	0.22 ± 0.91
14 years^d^	0.14 ± 1.10	0.12 ± 1.06	0.27 ± 1.18	0.004 ± 1.09	0.23 ± 1.20
Δ from 6 to 14 years^d^	−0.01 ± 0.80	−0.02 ± 0.78	0.10 ± 0.82	−0.11 ± 0.87	0.05 ± 0.83
ADHD traits score^e^, 6 years, mean ± SD, (scale: 0–12)	2.9 ± 2.5	1.7 ± 1.4	6.1 ± 1.4	2.5 ± 1.3	7.0 ± 1.9
Autistic traits score^e^, 6 years, mean ± SD, (scale: 0–26)	2.1 ± 2.4	1.1 ± 1.0	1.7 ± 1.0	5.1 ± 1.5	6.6 ± 3.1
Eating behaviours, 10 years, mean ± SD, (scale: 1–5)					
Food responsiveness^f^	1.8 ± 0.8	1.8 ± 0.7	2.0 ± 0.8	1.8 ± 0.8	2.0 ± 0.9
Enjoyment of food^f^	3.6 ± 0.7	3.6 ± 0.7	3.6 ± 0.7	3.5 ± 0.8	3.4 ± 0.8
Emotional overeating^f^	1.5 ± 0.7	1.4 ± 0.6	1.6 ± 0.7	1.5 ± 0.7	1.7 ± 0.8
Emotional undereating^f^	2.2 ± 0.9	2.2 ± 0.9	2.3 ± 0.9	2.3 ± 1.0	2.5 ± 1.0
Satiety Responsiveness^f^	2.6 ± 0.7	2.6 ± 0.6	2.6 ± 0.7	2.6 ± 0.7	2.7 ± 0.7
Picky eating^g^	2.3 ± 0.9	2.2 ± 0.9	2.4 ± 0.9	2.6 ± 0.9	2.6 ± 0.9
**Mother**					
Age at inclusion (years), mean ± SD	31.6 ± 4.5	32.8 ± 4.4	31.2 ± 4.9	31.7 ± 4.1	30.5 ± 5.2
Educational level (%)					
High	2470 (57.3)	1715 (61.1)	299 (47.3)	282 (61.8)	174 (41.2)
Middle	1258 (29.2)	771 (27.5)	215 (34.0)	126 (27.6)	146 (34.8)
Low	586 (13.6)	320 (11.4)	118 (18.7)	48 (10.5)	100 (23.8)
Psychopathology^h^, mean ± SD, (scale: 0 to 120)	12.1 ± 15.4	10.4 ± 13.4	13.5 ± 15.5	12.3 ± 14.6	21.7 ± 23.1

^a^ Values not imputed for main variables of interest (ADHD and autistic traits score, eating behaviours and BMIz at 14 years).

^b^ ADHD_High_: ≥80th percentile on ADHD traits and <80th percentile on autistic traits; ASD_High_: <80th percentile on ADHD traits and ≥80th percentile on autistic traits); ADHD + ASD_High_: ≥80th percentile on ADHD and autistic traits; REF, <80th percentile on both ADHD and autistic traits.

^c^ Sex‐ and age‐adjusted Body Mass Index (BMI) z score calculated using Dutch Reference growth curves.

^d^ *n* = 3495.

^e^ Child Behaviour Checklist (CBCL)/1.5–5.^20^

^f^ Children's Eating Behaviour Questionnaire.^13^

^g^ Stanford Feeding Questionnaire.^29^

^h^ Brief Symptom Inventory.^34^

### 3.1 Aim 1

Multiple linear regression models for the effect of ADHD and autistic traits subgroup on eating behaviours are shown in Table 2. Children in the ADHD + ASD_High_ group showed increased scores across all eating behaviours compared to the REF group, except for enjoyment of food where they had a relatively lower score. Children in the ADHD_High_ group showed greater food responsiveness, emotional overeating, emotional undereating and picky eating scores relative to the REF group. Children in the ASD_High_ group showed greater food responsiveness, emotional undereating and picky eating scores relative to the REF group. In addition, the ASD_High_ group had lower food responsiveness scores compared to the ADHD_High_ and ADHD + ASD_High_ groups; and lower emotional overeating compared to the ADHD + ASD_High_ group. The ADHD_High_ group had significantly lower estimates for picky eating compared to the ASD_High_ and ADHD + ASD_High_ groups. Only one sex interaction was observed for the ASD_High_ subgroup on enjoyment of food (*p* = 0.046). Boys in the ASD_High_ subgroup had a lower enjoyment of food compared to the REF subgroup (*β* = −0.18; 95% CI: −0.32, −0.05, *p* < 0.01) and this association was not significant in girls (*p* = 0.95). The independent effects of ADHD and autistic traits as continuous variables on eating behaviours are shown in Table S2.

**TABLE 2:** Multiple linear regression analysis showing the association between ADHD and autistic traits subgroup and eating behaviour, and BMIz

Outcome	REF *n* = 2806 (65.0%)	ADHD_High_ *n* = 632 (14.6%)	ASD_High_ *n* = 456 (10.6%)	ADHD + ASD_High_ *n* = 420 (9.7%)
	β (95% CI)			
Eating behaviour – 10 years (*n* = 4314)				
Food responsiveness	—	0.34 (0.26, 0.42)***	0.12 (0.02, 0.21)*	0.31 (0.21, 0.41)***
Enjoyment of food	—	0.01 (−0.07, 0.10)	−0.10 (−0.20, −0.01)^a^	−0.16 (−0.26, −0.05)**
Emotional overeating	—	0.18 (0.09, 0.26)***	0.03 (−0.06, 0.13)	0.27 (0.17, 0.38)***
Emotional undereating	—	0.18 (0.10, 0.27)***	0.17 (0.07, 0.27)***	0.32 (0.21, 0.42)***
Satiety responsiveness	—	0.07 (−0.01, 0.15)	0.08 (−0.01, 0.18)	0.11 (0.01, 0.21)*
Picky eating	—	0.11 (0.03, 0.20)**	0.33 (0.23, 0.43)***	0.34 (0.23, 0.44)***
BMIz – 14 years (*n* = 3495)	—	0.09 (0.01, 0.16)*	−0.07 (−0.16, 0.01)	0.01 (−0.10, 0.10)

^a^ Significant sex interaction. ADHD_High_: >80th percentile on ADHD traits and < 80th percentile on autistic traits; ADHD_High_: ≥80th percentile on ADHD traits and < 80th percentile on autistic traits; ASD_High_: <80th percentile on ADHD traits and ≥ 80th percentile on autistic traits); ADHD+ASD_High_: ≥80th percentile on ADHD and autistic traits; REF, <80th percentile on both ADHD and autistic traits. ADHD and autistic traits assessed with the Child Behaviour Checklist (CBCL)/1.5–5.^20^ Each model adjusts for the child's sex, ethnicity, birth weight, BMIz at 6 years, ADHD medication and maternal age at recruitment, education and psychopathology.

* *p* < 0.05.

** *p* < 0.01.

*** *p* < 0.001 (after correction for multiple testing).

### 3.2 Aim 2

Children with complete BMIz data at 14 years were included in the second analysis (*n* for each group: ADHD_High_ = 517, ASD_High_ = 360, ADHD+ASD_High_ = 318 and REF = 2300). A slightly higher proportion of BMIz data at 14 years were available for children in the REF and ADHD_High_ groups (82% for each group) compared to the ASD_High_ (79%) and ADHD + ASD_High_ (76%) groups (*χ* ^2^[3] = 10.85, *p* = 0.01). Children in the ADHD_High_ group had a greater BMIz at 14 years relative to the REF group (Table 2). There were no other differences in BMIz scores between subgroups and there were no subgroup × sex interactions on BMIz. Therefore, only the ADHD_High_ and REF subgroups were examined as independent variables in the mediation analysis. In multiple linear regression analyses, BMIz at 14 years was positively associated with food responsiveness, enjoyment of food and emotional overeating, and negatively associated with satiety responsiveness (Table S3). Only food responsiveness and emotional overeating were examined as mediators in the exploratory analysis because these variables were associated with both the independent (REF vs. ADHD_High_) and outcome variables (BMIz at 14 years).

**FIGURE 1:**
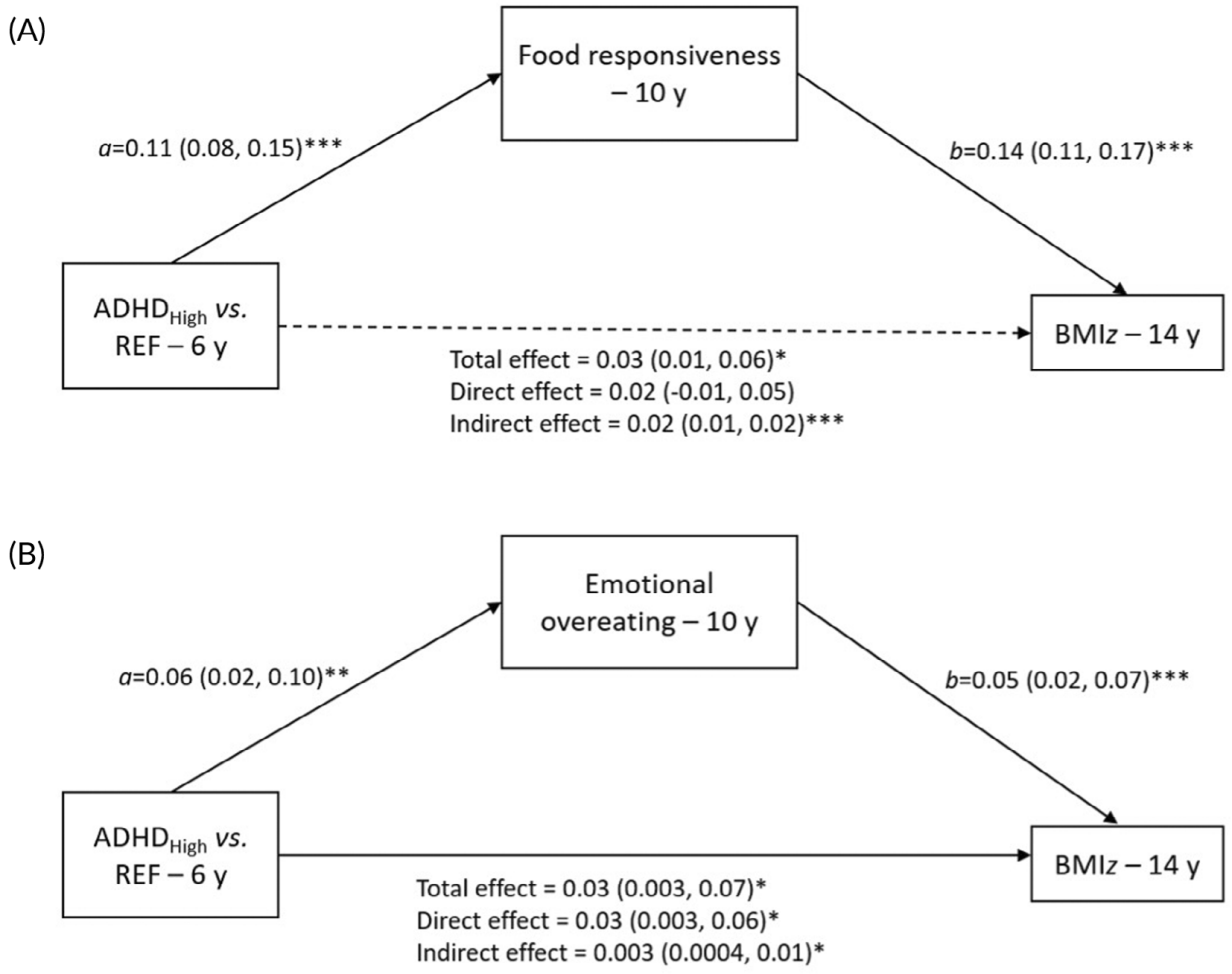
Mediation model showing the association of between the ADHD_High_ (*n* = 517) versus REF (*n* = 2300) subgroup on BMIz indirectly through (A) food responsiveness and (B) emotional overeating. β (95% CI) is shown for each pathway; solid black lines represent statistically significant pathways (*p < 0.05; **p < 0.01; ***p < 0.001); dotted lines represent non‐significant pathways. Model adjusts for the child's sex, ethnicity, birth weight, BMIz at 6 years, ADHD medication and maternal age at recruitment, education and psychopathology

In the food responsiveness mediation model (Figure 1A), the indirect effect of belonging to the ADHD_High_ (vs. REF) subgroup on BMIz through food responsiveness was statistically significant (indirect effect: *β* = 0.016; 95% CI: 0.010, 0.022, *p* < 0.001). The direct effect of ADHD_High_ (vs. REF) on BMIz was no longer significant once accounting for the indirect effect of food responsiveness (direct effect: *β* = 0.017; 95% CI −0.010, 0.0455, *p* = 0.22), suggesting full mediation through food responsiveness. In the emotional overeating mediation model (Figure 1B), there was also a small but significant indirect effect of belonging to the ADHD_High_ (vs. REF) subgroup on BMIz through emotional overeating (indirect effect: *β* = 0.003; 95% CI: 0.0004, 0.01, *p* < 0.05). The direct effect of ADHD_High_ (vs. REF) on BMIz remained significant when accounting for the indirect effect of emotional overeating (direct effect: *β* = 0.030; 95% CI: 0.003, 0.057, *p* < 0.05), suggesting partial mediation through emotional overeating.

Rerunning the analyses without imputing missing values on covariates produced similar results.

## 4 DISCUSSION

This study examined how ADHD and autistic traits in the general paediatric population relate uniquely and combined to various eating behaviours and weight. Results show that children with elevated ADHD and autistic traits scores (ADHD + ASD_High_) at 6 years expressed more ‘extreme’ eating behaviours at 10 years than the referent children (REF), showing both more food approach (i.e., increased food responsiveness and emotional overeating) and more avoidant behaviours (i.e., increased emotional undereating, satiety responsiveness/slowness in eating and picky eating, and decreased enjoyment in food). Regarding unique effects, children with high autistic traits only (ASD_High_) generally expressed food avoidant behaviours more frequently, while children with high ADHD traits only (ADHD_High_) generally expressed food approach behaviours more frequently. Children with high ADHD traits (ADHD_High_) had a greater BMIz score at 14 years relative to the referent children, and this association was mediated by two food approach behaviours: food responsiveness and emotional overeating. Regardless of associations with weight, extreme levels of food approach and/or avoidant eating behaviours may require parents to redirect children's focus on eating, coax them to eat or manage disruptive behaviour during mealtimes, thus impacting the family's mealtime experience.^38^ Yet, managing eating challenges is rarely addressed in early developmental interventions^39^ and is under‐recognized by health professionals.^40^ Findings from the current study suggest that targeted, population‐wide lifestyle interventions could benefit from screening for elevated ADHD and autistic traits, and tailoring strategies based on children's neurodevelopmental profile.

Studies have rarely investigated the co‐occurrence of elevated ADHD and autistic traits, and their potential shared obesity risk factors. In our population‐based cohort, children with the high‐level ADHD and autistic trait phenotype (ADHD + ASD_High_) had a unique eating profile suggesting both avid and restricted appetites. This is difficult to interpret, because many of the food approach dimensions tend to inversely correlate with food avoidant dimensions on the CEBQ.^13^ Nevertheless, this seemingly contradictory appetitive profile observed in children with the ADHD and autistic trait phenotype may indicate dysregulated eating that could focus food approach behaviours on a restricted number of lower‐quality foods.^10^, ^11^ Their expression of eating behaviours may be contingent on the food type and amount offered or available, the social and emotional mealtime context, and their parents' interpretation of their behaviour. Overlapping features of ADHD and ASD, such as inattention, impulsivity and impaired effortful control, could limit children's capacity to maintain focus during mealtimes, perhaps presenting as food avoidant behaviour.^41^ Conversely, these overlapping features may also underlie the expression of food approach behaviours; for example, difficulty in resisting palatable food when it is available. Our results also show some evidence of a small ‘additive’ effect of elevated ADHD and autistic traits on emotional over‐ and under‐eating (which are highly correlated), where effect estimates were approximately double those of the ASD_High_ and ADHD_High_ subgroups. High negative affect and emotion dysregulation are common in ADHD and ASD,^41^ and the presence of both emotional over‐ and under‐eating may indicate deficits in self‐regulation or recognizing internal feelings of hunger and fullness.^42^ Supporting children with elevated ADHD and autistic traits in adopting more adaptive coping strategies could therefore form a fundamental part of future interventions. Engaging parents of children with elevated ADHD and autistic traits in qualitative research could provide a deeper insight into the contextual mealtime processes and nuanced characteristics of food approach and avoidant behaviours. Such insight could contribute to the co‐creation of supportive family feeding interventions to assist parents in identifying and sensitively responding to their child's individual behavioural, sensory and nutritional needs. As self‐regulation may be impaired in these children, interventions that also facilitate parents' adoption of coping strategies at mealtimes that promote child–parent co‐regulation will be required.

Findings from the current study replicate well‐documented clinical associations between ADHD and increased risk of overweight^43^ in a general paediatric population. We also enrich the understanding of this known association by demonstrating that indicators of a heartier appetite, such as food responsiveness and emotional overeating, may play a role in increased BMIz for children with high‐level ADHD traits (ADHD_High_). The scant literature available examining relationships between ADHD traits, eating behaviours and BMI suggests that ADHD traits may precede changes in eating and BMI.^10^, ^32^, ^44^ For example, a community‐based sample of 4 year old children (*n* = 100) showed that ADHD traits such as inattention and/or hyperactivity predicted increases in food responsiveness, emotional overeating and BMIz score at 7 years, while the reverse association was not significant.^44^ In the current study, the separate mediation analyses suggested that food responsiveness may have a more salient role in the association between high‐level ADHD traits and BMI compared to emotional overeating; 48% of the total effect of ADHD_High_ (vs. REF) on BMI was explained indirectly through food responsiveness, while 9% was explained indirectly through emotional eating. This is perhaps unsurprising, as food responsiveness is associated with a greater preference for^45^ and intake of^46^ energy‐dense and nutrient‐poor foods, and more frequent meal consumption,^47^ and is more strongly associated with BMI compared to emotional overeating in children.^12^ Together, the results from our mediation analyses suggest that reducing opportunities for children to engage in overeating may be an effective target for weight management in children with high‐level ADHD traits. Nevertheless, these eating behaviours could also be precursors to disinhibited eating tendencies that link ADHD (symptoms) to disordered eating behaviours such as loss of control eating^48^ or binge eating.^49^ It is worth noting that eating behaviours may be one of many complex mechanisms underlying the association between ADHD and weight. Overall, findings from the current study suggest that preventative obesity work may be particularly needed for children with elevated ADHD traits.

Children with high‐level ASD traits (ASD_High_) tended to show more food avoidance, with greater picky eating, emotional undereating and lower enjoyment of food (in boys only) compared to children in the referent group. In addition, we found that children in the ASD_High_ subgroup were reported to be pickier eaters but less food responsive than the ADHD_High_ subgroup. Taken together, these tendencies may be attributed to eating phenotypes previously reported in children diagnosed with ASD, such as restricted food preferences and dietary intake (i.e., food selectivity), and also a tendency to overeat palatable, well‐liked foods (i.e., selective overeating).^17^ Compared to neurotypical children, children with ASD have a lower dietary variety,^50^ and consume fewer fruits and vegetables, and more sugar‐sweetened beverages and snacks.^51^ Therefore, children with subclinical but elevated autistic traits who approach the same, energy‐dense and nutrient‐poor foods may be at risk of poor nutritional status.^11^ Previous research from the current cohort showed that autistic traits, measured via the Social Responsiveness Scale,^25^ was positively associated with food responsiveness, picky eating and emotional overeating.^33^ In addition, stronger associations between autistic traits and emotional over‐ and under‐eating were found in girls.^33^ These findings are not necessarily comparable to the main findings in the current study, probably due to accounting for ADHD in the subgroup analyses. The current findings suggest that children with elevated autistic traits may require different strategies that target distinct eating behaviours compared to those with high‐level ADHD traits only, such as repeated exposure to promote the acceptance of healthy foods.

### 4.1 Strengths and limitations

The study findings must be interpreted considering some limitations. The use of parent‐reported measures may introduce shared‐reporter and social desirability bias. The CBCL^20^ was used to obtain parental reports of ADHD traits and autistic traits, which is primarily used as a screening tool for various DSM‐oriented conditions and therefore may not necessarily capture the extreme lower‐end of the ADHD and ASD continuum. Subgroups were determined using theoretically‐ and developmentally‐informed sample‐based cut‐offs rather than clinical cut‐offs. Although this approach has been undertaken previously and helps capture children who present as sub‐clinical in early life in parental report but later meet diagnostic criteria,^22^ some caution is needed when generalizing to other population‐based samples. Moreover, because we included subclinical ADHD and autistic traits, and our findings may not be accurate for to children with ADHD and ASD diagnoses and more severe symptomatic presentations. We also acknowledge that ADHD and ASD represent heterogeneous conditions that are difficult to quantify, and more research into sub‐phenotypes is required. Furthermore, sociodemographic differences between included participants and those who were excluded due to missing data may limit the generalizability of findings to lower socioeconomic populations. Finally, we did not test the directionality of associations between variables. Longitudinal research comprising of multiple, repeated measures (ideally from a young age) is required to determine the direction of effects between variables.

Strengths of the study include a large, population‐based sample. Unlike previous studies, we attempted to disentangle ADHD and autistic traits to examine their combined and unique associations with multiple eating behaviours. We also tested potential mediators of the association between ADHD and autistic traits subgroup and BMI. Future research could employ data‐driven approaches to elucidate how ADHD and autistic trait phenotypes in childhood is associated with stable, trait‐based eating profiles. In addition, intensive longitudinal methods (like ecological momentary assessment) could be used to investigate the association between ADHD and autistic traits, and variability of eating behaviours at a state‐based level (e.g., emotional over‐ and under‐eating). Future research could also explore different developmental stages, as associations with BMI may not emerge until a later age, particularly as adolescents become more autonomous in eating.

ADHD and ASD have overlapping features^6^, ^7^, ^8^ but are rarely studied together in public health‐and obesity prevention‐related research. Results from the current population‐based study suggest that children's elevated ADHD and/or autistic phenotypes show distinctive associations with eating behaviours and BMIz score relative to children with low‐level ADHD and autistic traits. Many of the eating behaviours associated with ADHD and autistic traits in the current study influence energy intake,^47^ diet quality^52^ or BMI.^12^ Therefore, early screening for elevated ADHD and autistic traits could be considered when implementing lifestyle or developmental interventions, to optimize the delivery of tailored nutrition advice or obesity‐prevention strategies. Educating health professionals on how to address the feeding concerns of parents of children with elevated ADHD or autistic traits could also create a conducive therapeutic environment.^40^ Identifying sensitive strategies to support caregivers in responsively feeding children with neuroatypical traits will be critical to enhance the efficacy of interventions, and promote positive family mealtime interactions.

**List of Abbreviations**
ADHD: Attention‐Deficit/Hyperactivity Disorder
ADHD+ASD_High_
: subgroup with ≥80th percentile scores on ADHD and autistic traits
ADHD_High_
: subgroup with ≥80th percentile score on ADHD traits and <80th percentile score on autistic traits
ASD: Autism Spectrum Disorder
ASD_High_
: subgroup with <80th percentile score on ADHD traits and ≥ 80th percentile score on autistic traits
BMI: Body Mass Index
CBCL: Child Behaviour Checklist
CEBQ: Children's Eating Behaviour Questionnaire
CPRS‐R:S: Parent Rating Scale–Revised Short Form
DSM: Diagnostic and Statistical Manual of Mental Disorders
REF: <80th percentile score on both ADHD and autistic traits

## AUTHOR CONTRIBUTIONS

Holly A. Harris, April Bowling and Pauline W. Jansen conceived the research; Holly A. Harris performed the statistical analyses; Holly A. Harris, April Bowling and Pauline W. Jansen interpreted the results and wrote the paper; Susana Santos and Kirstin Greaves‐Lord critically reviewed the manuscript for important intellectual content; Holly A. Harris has primary responsibility for the final content. All authors have read and approved the final manuscript.

## CONFLICT OF INTEREST

No conflict of interest was declare.

## Supporting information


**Data S1** Supporting inforamtion.
