# Supplementary material for: Child ADHD and autistic traits, eating behaviours and weight: A population‐based study
Source: Pediatr Obes. 2022 Jun 24;17(11):e12951. doi: 10.1111/ijpo.12951 (PMC9786764; doi:10.1111/ijpo.12951)
Supplement: Supplementary file 1 — Data S1 Supporting inforamtion. [file IJPO-17-e12951-s001.pdf]

Supplementary file

Figure S1. Participant flow diagram

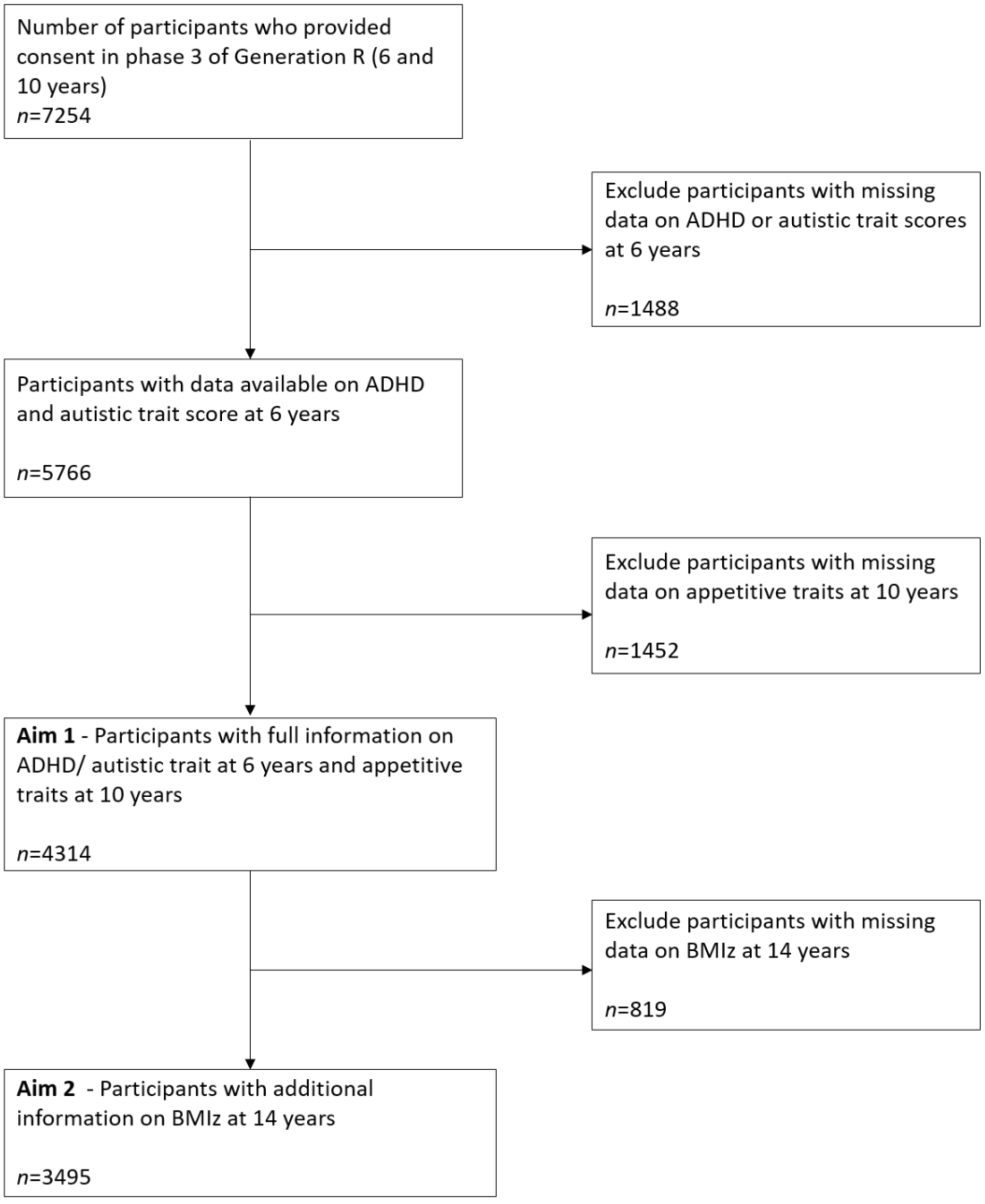

Table S1. Non-imputed population characteristics

**Child**	All<br>*n*=4314	N available	REF<br>*n*=2806<br>(65.0%)	$ADHD_{High}$<br>*n*=632 (14.6%)	ASD_{High}<br>*n*=456 (10.6%)	$ADHD+ASD_{High}$<br>*n*=420 (9.7%)
Sex		4313				
Boys, (%)	2141 (49.6)		1269 (45.2)	354 (56.0)	258 (56.6)	260 (61.9)
Birth weight (g), mean ± SD	3431.2 ± 571.3		3448.0 ± 562.0	3391.8 ± 573.8	3448.5 ± 569.1	3359.5 ± 623.4
Ethnicity		4312				
Western ethnicity (%)	3386 (78.5)		2265 (80.8)	478 (75.6)	371 (81.4)	272 (64.8)
ADHD medication						
Yes, (%)	136 (3.3)	4135	46 (1.7)	42 (7.0)	10 (2.3)	38 (9.6)
BMIz[a], mean ± SD						
6y	0.19 ± 0.87	4103	0.18 ± 0.85	0.24 ± 0.92	0.13 ± 0.90	0.21 ± 0.92
**Mother**						
Age at inclusion (years), mean ± SD	31.6 ± 4.6	4313	31.8 ± 4.4	31.2 ± 4.9	31.7 ± 4.1	30.5 ± 5.2
Educational level (%)		4110				
High	2370 (57.7)		1650 (61.6)	286 (47.0)	269 (62.6)	165 (41.8)
Middle	1184 (28.8)		723 (27.0)	208 (34.2)	117 (27.2)	136 (34.4)
Low	556 (13.5)		304 (11.4)	114 (18.8)	44 (10.2)	94 (23.8)
Psychopathology, mean ± SD, scale: 0 to 120	12.1 ± 15.6	3329	10.3 ± 13.6	13.7 ± 15.6	13.0 ± 15.8	21.8 ± 23.2

[a]Sex- and age-adjusted Body Mass Index (BMI) score calculated using Dutch Reference growth curves; $ADHD_{High}$: >80th percentile on ADHD traits and <80th percentile on autistic traits; ASD_{High}: <80th percentile on ADHD traits and >80th percentile on autistic traits); $ADHD+ASD_{High}$: >80th percentile on ADHD and autistic traits; REF, <80th percentile on both ADHD and autistic traits.

Table S2. Pooled estimates of multiple linear regression analyses examining independent associations between ADHD and autistic traits at 6 years and eating behaviors at 10 years (*n*=4314)

	**Outcome** β (95% CI)					
**Predictor(s)**	Food responsiveness	Enjoyment of food	Emotional overeating	Emotional undereating	Satiety Responsiveness	Picky eating
Model 1						
ADHD traits	0.18 (0.15, 0.21)***	-0.04 (-0.07, -0.004)*	0.10 (0.07, 0.14)***	0.12 (0.08, 0.15)***	0.05 (0.02, 0.08)**	0.09 (0.06, 0.13)***
Model 2						
Autistic traits	0.11 (0.08, 0.14)***	-0.07 (-0.10, -0.04)***	0.08 (0.05, 0.11)***	0.11 (0.08, 0.14)***	0.04 (0.01, 0.07)*	0.15 (0.12, 0.18)***
Model 3						
ADHD traits	0.15 (0.12, 0.19)***	-0.01 (-0.04, 0.03)	0.09 (0.05, 0.12)***	0.08 (0.05, 0.12)***	0.04 (0.01, 0.07)*	0.04 (0.003, 0.07)*
Autistic traits	0.05 (0.02, 0.08)**	-0.06 (-0.10, -0.03)***	0.05 (0.01, 0.08)**	0.08 (0.04, 0.11)***	0.02 (-0.01, 0.06)	0.13 (0.10, 0.17)***

*p<0.05, **p<0.01, ***p<0.001, All models adjust for the child's sex, ethnicity, birth weight, BMIz at 6 years, ADHD medication and maternal age at recruitment, education and psychopathology. ADHD and autistic traits assessed with the Child Behavior Checklist (CBCL)/1.5-5[20]; Eating behaviors measured using the Children's Eating Behaviour Questionnaire[13] and the Stanford Feeding Questionnaire[29]

Table S3. Pooled estimates of multiple linear regression analyses showing the association between eating behaviors and BMIz (*n*=3495)

	BMIz – 14 years
**Eating behavior – 10 years**	β (95% CI)
Food responsiveness	0.16 (0.13, 0.19)***
Enjoyment of food	0.06 (0.03, 0.08)***
Emotional overeating	0.06 (0.03, 0.09)***
Emotional undereating	-0.01 (-0.03, 0.02)
Satiety responsiveness	-0.07 (-0.10, -0.04)***
Picky eating	0.01 (-0.02, 0.03)

*p<0.05, **p<0.01, ***p<0.001. Models adjust for the child's sex, ethnicity, birth weight, BMIz at 6 years, ADHD medication and maternal age at recruitment, education and psychopathology. Eating behaviors measured using the Children's Eating Behaviour Questionnaire[13] and the Stanford Feeding Questionnaire[29]
